# Use of surgical videos available on social media among retina surgeons: results of a vitreoretinal specialist survey

**DOI:** 10.5935/0004-2749.2022-0166

**Published:** 2023-03-08

**Authors:** Luiz Filipe Adami Lucatto, Gabriel Castilho Sandoval Barbosa, Juliana Moura Bastos Prazeres, Emmerson Badaró Cardoso, Ricardo Luz Leitão Guerra, Luiz Henrique Soares Gonçalves de Lima, Eduardo Büchele Rodrigues

**Affiliations:** 1 Ophthalmology Department, Universidade Federal de São Paulo, São Paulo, SP, Brazil; 2 Ophthalmology Department, Instituto Suel Abujamra, São Paulo, SP, Brazil; 3 Ophthalmology Department, Obras Sociais Irmã Dulce, Salvador, BA, Brazil; 4 Ophthalmology Department, Saint Louis University Eye Institute, St. Louis, MO, USA

**Keywords:** Learning, Education, distance, Social media, Vitreoretinal surgery, Surgeons, Surveys & questionnaires, Aprendizagem, Educação a distância, Mídias sociais, Cirurgia vítreo-retiniana, Cirurgiões, Inquéritos e questionários

## Abstract

**Purpose:**

This study aimed to assess and interpret how vitreoretinal surgeons use surgical videos available on social media as complementary learning tools to improve, review, or update their abilities, considering their different levels of expertise.

**Methods:**

In this cross-sectional survey, an online survey was sent to vitreoretinal specialists and fellows.

**Results:**

This survey included 258 participants, of whom 53.88% had been in practice for >10 years (senior surgeons), 29.07% between 4 and 10 years (young surgeons), and 17.05% for <3 years (surgeons in training). Retinal surgical videos available on social media were used by 98.84% of the participants (95% confidence interval, 97.52%-100%). YouTube (91%) was the most common source of videos, and surgeons in training watched more videos on YouTube than senior surgeons. Regarding the preferred method when preparing for a procedure, 49.80% of the participants watched surgical videos available on social media, 26.27% preferred to “consult colleagues”, and 18.82% preferred to seek information in scientific articles. Participants valued the most the “image quality” (88%) and presence of “surgical tips and tricks” (85%).

**Conclusion:**

Surgical videos can provide benefits in acquiring strategic skills, such as decision-making, surgical planning, and situational awareness. Retina surgeons used them as teaching aids regardless of their level of expertise, despite being relatively more valuable to surgeons in training or young surgeons.

## Abstract (pt)

**Objetivo:**

Avaliar e interpretar como os cirurgiões vitreorretinianos utilizam os vídeos cirúrgicos disponíveis nas mídias sociais como ferramentas complementares de aprendizagem para melhorar, revisar ou atualizar suas habilidades, considerando seus diferentes níveis de especialização.

**Métodos:**

Nesta pesquisa transversal, um survey online foi enviado à especialistas e aprendizes na área vítreo-retiniana.

**Resultados:**

Esta pesquisa incluiu 258 participantes, dos quais 53,88% atuavam há mais de 10 anos (cirurgiões seniores), 29,07% entre 4 e 10 anos (cirurgiões jovens) e 17,05% há menos de 3 anos (cirurgiões em treinamento). Vídeos cirúrgicos de retina nas mídias sociais foram usados por 98,84% dos participantes (intervalo de confiança de 95%, 97,52%-100%). A fonte mais comum de acesso aos vídeos foi o YouTube (91%), e o grupo de cirurgiões com menos de 3 anos de experiência assistiu mais vídeos no YouTube em comparação aos cirurgiões seniores. Assistir a vídeos cirúrgicos nas redes sociais foi o método preferido na preparação para um procedimento para 49,80% dos participantes versus 26,27% que preferiram “consultar colegas” e 18,82% que preferiram buscar informações em artigos científicos. A “qualidade de imagem” (88%) e a presença de “dicas e truques cirúrgicos” (85%) foram as características dos vídeos mais valorizadas pelos participantes.

**Conclusão:**

O uso de vídeos cirúrgicos pode trazer benefícios na aquisição de habilidades estratégicas, como tomada de decisão, planejamento cirúrgico e consciência situacional. Sua aplicação como auxiliar de ensino foi utilizada por cirurgiões de retina independentemente de seu nível de especialização, apesar de ser relativamente mais valioso para cirurgiões em formação ou com menos de 10 anos de experiência.

## INTRODUCTION

In the 1920s, Jules Gonin, a Swiss ophthalmologist, recognized the role of retinal breaks in retinal detachment and proposed their sealing and drainage of subretinal fluid through the sclera^(1)^, which marked the beginning of the history of retinal surgery. Several techni-ques and procedures were then proposed, until Robert Machemer, regarded as the “Father of Modern Vitreoretinal Surgery”, introduced the concept of pars plana vitrectomy in 1971^(2)^. Along with this new concept, the first vitreoretinal surgical video^(3)^ of a diabetic vitreous hemorrhage was created in 1972-1973, in which Robert Machemer recorded and perpetuated the techniques used and the instruments he developed.

With the constant evolution of surgical techniques, several new approaches have been created and described, especially in books and journals, in scientific articles that feature illustrative descriptions. In association with scientific information, the direct and joint participation of both mentors and apprentices characterize the conventional surgical training method, which historically encompasses all medical subspecialties. However, the use of online resources represents an essential role in knowledge acquisition and has been gaining currency in surgical training^(4^,^5)^, mainly because it allows access to information quickly and inexpensively, breaking down geographic and time limitations.

Surgical video recordings may be safe and effective teaching tools in acquiring crucial surgical and strategical skills, such as decision-making, surgical planning, situational awareness, and understanding of surgical steps^(6^,^7)^. Additionally, the reproduction of surgical procedures through edited video recordings and live surgeries has been gaining widespread popularity in ophthalmology because both motor and cognitive abilities can be acquired through observation^(8)^.

Social media is a mediated online technology that facilitates creation and sharing of information, ideas, and other forms of expression through virtual communities and networks^(9)^. Within the surgical training scenario, with rapid and constant changes in vitreoretinal surgeries, social media offers opportunities for teaching, training, research, and social interaction. Recent studies have reported the use of social media among retinal specialists^(10^,^11)^. However, information on how vitreoretinal surgeons and fellows have used surgical video recordings as a complementary teaching aid in the field is lacking.

This study aimed to evaluate and interpret how vitreo-retinal surgeons and fellows use surgical videos available on social media as a complementary learning tool to improve, review, or update their abilities, considering their different levels of expertise.

## METHODS

In this cross-sectional survey study, an online survey was sent to vitreoretinal specialists and fellows using Google Forms. This was conducted to evaluate how retina surgeons use the surgical videos available on social media as a learning tool. Retinal specialists and fellows registered in medical societies were invited to participate in the study via email and WhatsApp messages. Because validated questionnaires involving the parameters of interest for this study are not available and no questionnaire has already been applied to a sample from another surgical medical area^(4^,^5)^, an adapted version of a questionnaire was generated and applied to vitreoretinal specialists and fellows. After reviewing the literature and remote discussions, the most appropriate questions for the assessment of the use of retinal surgical videos available on social media for surgery preparation and medical updating were defined.

The initial study questionnaire was reviewed by four retina surgeons and investigators and tested and modified by four retina surgeons and four fellows who volunteered to participate as a pretest group. During the pretest, the adequacy of each question was assessed, and poorly formulated questions and response options were identified and corrected. The questionnaire was also shortened to decrease fatigue and improve the overall style according to guidelines for conducting and reporting survey research^(12^,^13)^.

The final survey questionnaire was organized in two parts and comprised of four questions about the demographic characteristics of the participants and 11 questions about their attitudes toward the use of surgical videos available on social media as complementary educational tools in vitreoretinal surgeries. The questions were presented as multiple choice, checkboxes, linear scale, and open questions. The data collected detailed the use of surgical videos among the participants, frequency of access and topics, and characteristics of interest. The questionnaire also asked whether the participants used videos for surgery preparation and about the benefits and limitations of their use. The questionnaire was tested in a pilot study with the principal investigator and collaborators and reviewed before distribution.

Previous studies have typically reported a 30% response rate to online surveys^(14^,^15)^. Thus, a sample of >600 individuals was prespecified for this study to ensure a 30% response rate, and the proportion of respondents who use videos available on social media was estimated using a 95% confidence interval (CI) ± 2%. The questionnaires were distributed securely via weekly email and WhatsApp messages between May 1 and June 30, 2021. All responses were received anonymously and stored in a password-protected account on the server used to generate the survey. Two authors (LFAL and JMBP) analyzed the results.

The research ethics committee approved this study, and participants signed a consent form before participation through an electronic platform inserted at the beginning of the questionnaire. The authors maintained the confidentiality of the collected data.

Data analysis was performed using Stata/SE 12.0 software (StataCorp LLC, College Station, TX, USA). Descriptive statistics were expressed as frequency (n) and percentages (%) of categorical variables and mean or median (standard deviation, range) for continuous and ordinal variables. Pearson’s chi-square test (χ^^2^^) and Fisher’s exact test were used to verify differences between groups. The analysis of variance with the Bonferroni test was used to assess intergroup differences. All p-values <0.05 were indicated significance.

## RESULTS

**Figure 1:**
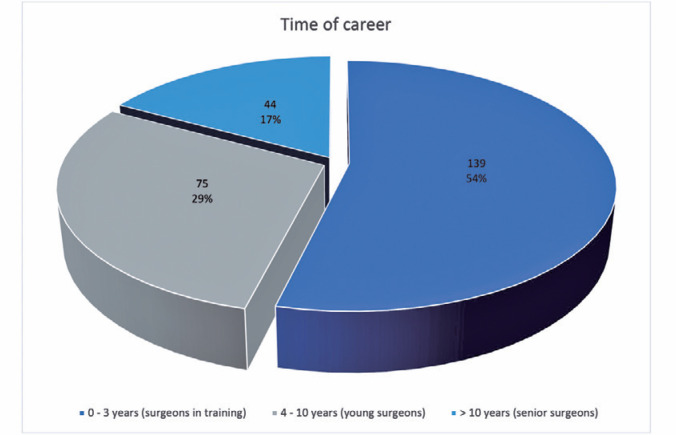
Distribution of the participants based on the length of practice.

Of the 806 questionnaires sent, 258 were returned (response rate, 32%). The mean respondent age was 41.12 (27-75) years. Of the participants, 53.88% had been in practice for >10 years (senior surgeons), 29.07% between 4 and 10 years (young surgeons), and 17.05% for <3 years (surgeons in training). Figure 1 shows data by the length of career.

In this study, 47 (18.22%) participants were attending a surgical training program (fellow), 55.31% were working in private clinics and hospitals, and 44.69% were working in universities. Most fellows (57.44%) reported having 2-5 surgical mentors in their training program. Only 0.04% of fellows trained with one mentor.

Moreover, 98.84% of our sample (95% CI, 97.52%-100.00%) used retinal surgical videos available on social media (Table 1). Only three participants had never watched surgical videos on these platforms: two of them did not use social media and another was not interested in this tool. This result did not reach significance when stratified by career stage (p=0.584, Fisher’s exact test).

**Table 1:** Analysis of social media accessed considering career stages

	Career stage			
	0-3 years	4-10 years	>10 years	Total
	n=44	n=75	n=139	n=258
**Have you watched retinal surgical videos posted on social media?**				
Yes	44 (100)	75 (100)	136 (97.84)	255 (98.84)
**Which social media sites do you use?**				
YouTube	100^*^	92	88	91
Webpages from retinal societies	64	73	84^*^	77
Instagram	91	88	51^*^	69
Facebook	23	21	18	20
Vimeo	27^*^	57	51	49
Twitter	7	4	4	4
Paid content	9	9	13	11
Eyetube	6	6	8	7

Results are presented as n (%).

YouTube (91%) was the most common source of videos, followed by websites of medical societies (BRAVS, ASRS, EURETINA, etc.; 77%), Instagram (69%), and Facebook (20%). Only 11% of the participants reported access to paid surgical videos content. Table 1 shows a significant difference between the sources of access and groups based on career length. Surgeons in training watched more videos on YouTube than senior surgeons. This group also watched fewer videos on Vimeo than did the other two groups. By contrast, medical society websites were accessed more by senior surgeons than by surgeons in training. Senior surgeons also watched fewer videos on Instagram than the other two groups.

The participant’s accessed videos weekly (50.20%) and monthly (18.43%). Approximately 15% of the surgeons reported accessing surgical videos available on social media platforms. The analysis stratified by career stage is shown in table 2. In total, 224 (86.82%) surgeons reported having watched surgical videos in preparation for surgery. This frequency increased to 94.67% among young surgeons, and a significant difference reached (p=0.0148) when compared with the senior surgeons (81.29%). In addition, 49.80% of the participants preferred watching surgical videos available on social media when preparing for a procedure, 26.27% preferred consulting colleagues, and 18.82% preferred seeking information in scientific articles (Table 2). On a 5-point Likert scale, with 1 point indicating useless and 5 very useful, the mean values were 4.80, 4.76, and 4.49 among surgeons in training, young surgeons, and senior surgeons, respectively. The difference between the senior group and other surgeon groups reached significance (p<0.03).

**Table 2:** Analysis of the frequency and utility of surgical videos considering career stages

	Career stage			
	Surgeons in training (0-3 years) 44	Young surgeons (4-10 years) 75	Senior surgeons (>10 years) 139	Total 258
**How often do you watch retinal surgical videos on social media**				
Daily	12 (27.27)	16 (21.33)	11 (8.09)	39 (15.29)
Weekly	27 (61.36)	40 (53.33)	61 (44.85)	128 (50.20)
Monthly	4 (9.09)	13 (17.33)	30 (22.06)	47 (18.43)
Less than monthly	1 (2.27)	6 (8)	34 (25)	41 (16.08)
**Have you already used these videos for surgical preparation?**				
Yes	40 (90.91)	71 (94.67)	113 (81.29)^*^	224 (86.82)
**When you need help preparing for a surgical case, what do you prefer to do?**				
Watch surgical videos	26	43	58	127 (49.80)
Reading cientific papers	4	15	29	48 (18.82)
Reading booking references	1	1	4	6 (2.35)
Consult with peers	10	16	41	67 (26.27)

Results are presented as n (%).

**Figure 2:**
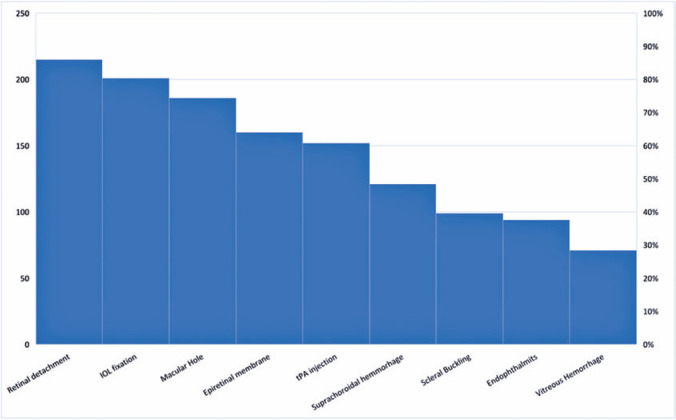
Surgical topics of greatest interest reported by the participants.

**Figure 3:**
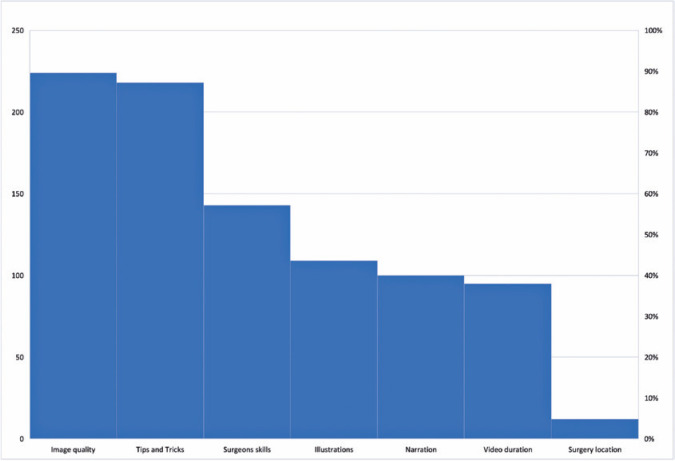
Features of retinal surgical videos that surgeons value the most.

The surgical topics of greatest interest were retinal detachment (84%), intraocular lens fixation (79%), and macular holes (73%). Endophthalmitis (37%) and vitreous hemorrhage (28%) were the topics of least interest (Figure 2). Regarding the video characteristics, 88% of the participants valued good-quality images. Moreover, 85% of the participants gave high importance to videos providing “tips and tricks.” The features that the surgeons valued the most in surgical videos are shown in figure 3.

## DISCUSSION

Ophthalmology is a visual medical specialty, and image-based learning is a coherent strategy. The retinal subspecialty has a vast array of surgical indications, which require different approaches and applicable techniques; therefore, online surgical videos may be important sources of complementary knowledge. During the past years, important changes in medical training have been observed. However, to the best of our knowledge, this is the first study to evaluate how vitreoretinal surgeons and fellows use surgical videos as complementary teaching aids.

As expected, the overwhelming majority of our sample (98.84%) watched retinal surgical videos available on social media to improve surgical techniques and knowledge. Data obtained were consistent with previous studies that have also included medical specialties other than ophthalmology^(4^,^5)^, indicating that the advent of information and communication technologies potentially improves surgical education through ease-of-access, hands-off media learning.

In this study, only three participants had never watched surgical videos available on social media platforms, do not use social media (two participants), or had no interest in this tool (one participant). The three participants were senior surgeons with at least 10 years of surgical experience; therefore, they likely have well--established surgical approaches and concepts that they have acquired conventionally. Despite this hypothesis, this result did not differ significantly (p=0.584, Fisher’s exact test) when stratified by career stage.

Regarding the type of social media that was used to access online videos, YouTube was the most common source, followed by websites of medical societies (BRAVS, ASRS, EURETINA, etc.), Instagram, and Facebook. These results are consistent with previous publications^(4^,^5)^. Surgeons in training watched more videos on YouTube (100%) than did senior surgeons (88%), and they prefer easy-access information tools with feedback, comments, and various approaches to a surgical technique. These findings suggested that younger surgeons place higher value on straightforward and instructive information to obtain views on each topic. However, only 11% of the participants reported access to paid surgical videos content, most of whom were senior surgeons. In addition, senior surgeons accessed retinal society webpages more often than the other two groups of surgeons. This suggested that more experienced surgeons value quality over quantity and use verified and validated information. This group also watched fewer videos on Instagram than did the other two groups of surgeons.

Regarding the frequency of access to online surgical videos, 50.2% of the participants watched videos weekly. The analysis stratified by career stage is shown in Table 2. Interestingly, most participants who answered “less than monthly” were senior surgeons (25%), whereas only 2.27% of the surgeons in training and 8% of the young surgeons chose this answer, indicating that younger surgeons access videos more often. These data were consistent with the participant responses when asked about their preferences when preparing for surgery. Of the 67 (26.27%) participants who preferred to “consult colleagues,” 41 (61.2%) were senior surgeons, implying that this group values sharing experiences among proficient professionals conventionally. However, the vast majority (49.8%) of the participants and participants when stratified preferred watching surgical videos available on social media when preparing for a procedure, which highlighted the importance of surgical videos in the retina subspecialty.

Participants were asked to stratify the usefulness of surgical videos as a complement to retinal teaching using a 5-point Likert scale, regarding acquiring, remembering, or recycling knowledge (Table 3). The mean values were 4.80, 4.76, and 4.49 among surgeons in training, young surgeons, and senior surgeons, respectively. A significant difference (p<0.03) was found between the senior group and the other two groups, indicating that surgeons in training resort to online information more often and place greater importance on this resource than experienced surgeons. This agrees with previous studies that included other medical specialties^(4)^.

**Table 3:** Analysis of the usefulness (acquiring, remembering, or recycling knowledge) of surgical videos considering career stages

How useful do you consider surgical videos on social media to complement teaching on retina (acquiring, remembering or recycling knowledge) 1 = not useful and 5 = very useful	
**Time of career**	Mean
0-3 years	4.80
4-10 years	4.76
>10 years	4.49^*^

The present study documented the areas of greatest interest and what participants value or seek during their online video searches (Figures 2 and 3). The participants searched for information about retinal detachment surgery most often, followed by intraocular lens fixation and macular hole. These themes were ranked in the first three of greatest interest by all participants because they are surgeries with several possible approaches and require surgical techniques of greater complexity, in addition to being major surgical indications in the retinal subspecialty. Moreover, participants value “image quality” and the availability of “surgical tips and tricks” the most. Because ophthalmology is primarily a visual specialty, exceptional image quality plays a fundamental role in knowledge sharing because it allows the spectator to clearly understand the steps and techniques in the video. By the same token, “tips and tricks” facilitate sharing of knowledge “outside the box”, which is often not found in scientific articles or books, and present techniques developed based on surgeons’ personal insights.

This study has some limitations. First, we did not compare the results from different geographic areas because only one participant in our sample lived outside Brazil. Second, there may be regional variations in training experience that this study could not discern. There may be regional variations and variations in training institutions (often dependent on the training stage) with different facilities, with subspecialties offering training, trainers, and clinical preceptors. Third, controlling for complex potential influences was beyond the scope of this study. Although many training issues are better addressed at a national level, we believe that this study provides valuable data to help understand how retina surgeons use online videos in practice.

As technological evolution facilitates access to qualified mentors, retina surgeons, specifically surgeons in training, can broaden their knowledge by watching surgical videos on the Internet, which facilitates both greater exposure to different surgical techniques, research for additional information in scientific articles, and discussions with more experienced colleagues. Videos can complement surgical learning as long as the content is educational and the videos are of good quality with detailed and pertinent explanations. The perception of the usefulness of the videos and critical analyses of the presence of inappropriate content can vary depending on the experience of the surgeons.

This study highlights the fundamental role of online surgical videos as complementary learning tools in the retina subspecialty. The results of this study suggested that retina surgeons, regardless of their level of expertise, use surgical videos as teaching aids, despite being relatively more valuable to surgeons in training or young surgeons.
